# Short Chain Fatty Acids (SCFA) Reprogram Gene Expression in Human Malignant Epithelial and Lymphoid Cells

**DOI:** 10.1371/journal.pone.0154102

**Published:** 2016-07-21

**Authors:** Lidiia Astakhova, Mtakai Ngara, Olga Babich, Aleksandr Prosekov, Lyudmila Asyakina, Lyubov Dyshlyuk, Tore Midtvedt, Xiaoying Zhou, Ingemar Ernberg, Liudmila Matskova

**Affiliations:** 1 Institute of Food Science and Technology, Kemerovo, Russia; 2 Department of Cell and Molecular Biology (CMB), Ludwig Institute for Cancer Research (LICR), Karolinska Institutet, Stockholm, Sweden; 3 Department of Microbiology, Tumor and Cell Biology (MTC), Karolinska Institutet, Stockholm, Sweden; University of South Alabama, UNITED STATES

## Abstract

The effect of short chain fatty acids (SCFAs) on gene expression in human, malignant cell lines was investigated, with a focus on signaling pathways. The commensal microbial flora produce high levels of SCFAs with established physiologic effects in humans. The most abundant SCFA metabolite in the human microflora is n-butyric acid. It is well known to activate endogenous latent Epstein-Barr virus (EBV), that was used as a reference read out system and extended to EBV+ epithelial cancer cell lines. N-butyric acid and its salt induced inflammatory and apoptotic responses in tumor cells of epithelial and lymphoid origin. Epithelial cell migration was inhibited. The n-butyric gene activation was reduced by knock-down of the cell membrane transporters MCT-1 and -4 by siRNA. N-butyric acid show biologically significant effects on several important cellular functions, also with relevance for tumor cell phenotype.

## Introduction

The metabolism of the human microbiota is intimately linked with that of the host, especially in mucosal tissues like the gut or the nasopharynx. A feature of the colonic microbiota metabolism is the fermentation of complex carbohydrates [1–3]. One important product of this metabolism is the production of short-chain fatty acids (SCFAs), which can have local effects at the site of production as well as systemic ones, through blood circulation [4–5].

SCFAs refer to free fatty acids with short (less than 6 carbons) aliphatic chains. They include formic acid, valeric acid, caproic acid and butyric acid and its structural isomers [6]. The SCFAs are taken up by blood and affect nutrition and the immune system [7]. N-Butyric acid is a 4-carbon straight chain SCFA, most interesting due to its high production by the microbiota. It reaches a concentration of 20mM in the colon. The metabolism of butyrate (salt of butyric acid) has been estimated to provide about 50% of the daily energy requirements of the gastrointestinal mucosa [8–9].

Although the establishment of a healthy gut microbiota, where bifido- and lactobacteria are prevailing, often coincides with an increase in butyrate concentration, neither lactobacilli nor bifidobacteria produce butyrate [10]. The majority of isolates producing high levels of butyrate (more than 10mM) are related to the Coccoides-Eubacterium phylae, which are other dominant members of the gut microbiota [11–12]. SCFAs are naturally found in foods as well. Thus, by modulation of a diet in favor of the proper microbiota one can modulate butyric acid levels locally and systemically [13].

Cells can be affected by SCFAs in three different ways. SCFA bind cell receptors that regulate cell proliferation and differentiation. SCFAs can enter cells through specific transporters and involve directly in the cellular metabolism, thus affecting cell energy status and signaling processes [14]. SCFAs can inhibit HDAC activity in the nuclei. All major SCFAs have HDAC inhibitory activity at large enough concentrations as shown in in vitro studies [15]. Inhibition of HDAC activity will promote access of transcription factors to promoters and activate gene expression. This, in turn can affect inflammatory and even carcinogenic processes at the gene-expression level [16–17].

We employed an Epstein-Barr virus (EBV) model system as a positive control in our study of effects of SCFAs on cells. More than 95% of adult human population carry EBV virus. It is well established that butyric acid can induce lytic EBV production and switch latency programs in EBV infected B cell lines [18]. Butyrate acts via histone deacetylation to induce lytic EBV replication and lysis of cells [19–21]. The first step of the switch from latency to the lytic virus cycle is the expression of immediate early transactivator genes, BZLF1 and BRLF1, which in concert, activate the subsequent viral lytic cascade [22–23]. The role of the nasopharyngeal microbiome and its metabolites for NPC-risk and–progression is will be of future major interest.

A panel of SCFAs focusing on butyric acid was tested. The expression of the specific transporters for butyric acid entry, MCT1 and 4 were investigated. Further genome-wide expression profiling of cells exposed to butyric acid was analyzed. Thus we could demonstrate a multifaceted effect of butyric acid involving several important host cell signaling pathways.

## Materials and Methods

### Chemicals

The SCFAs caproic, 2-ethylbutyric, n-butyric, isobutyric, isovaleric, n-valeric acids, were obtained from Merck (Darmstadt, Germany). Formic acid, sodium butyrate, hydrochloric acid were from Sigma-Aldrich (St. Louis, Missouri, USA). 12-*O*-Tetradecanoylphorbol-13-acetate (TPA) from Calbiochem (San Diego, California, USA) was used at 20 ng/ml.

### Cell lines and culture

C666-1[24], AGS[25], HONE1[26] and CNE2[27] are cell lines of human epithelial origin. C666-1 has been established from undifferentiated nasopharyngeal carcinoma (NPC). EBV latency II program is expressed in C666-1. AGS was established from gastric carcinoma and carry EBV in latency stage I. AGS cell line was kindly provided by professor Takada K., at University of Pittsburgh, USA. HONE1 and CNE2 are EBV negative cell lines. Nasopharyngeal carcinoma cell lines were kindly provided by professor Maria Li Lung’s laboratory at the University of Hong Kong, China. Raji and Rael are cell lines derived from Burkitt’s lymphomas and carry EBV genomes in latency type III and I, respectively [28,29]. BL cell lines we received from George Klein, MTC, KI. Raji and Rael cells were cultured in RPMI 1640 (HyClone ^™^, Thermo Scientific ^™^) containing 10% fetal bovine serum (HyClone ^™^, Thermo Scientific ^™^), penicillin (50 U/ml), streptomycin (50 U/ml) [29]. C666-1, HONE1, CNE2 and AGS cells were cultured in Iscove's Modified Dulbecco's Medium (HyClone ^™^, Thermo Scientific ^™^) prepared the same way but with15% FBS for the C666-1 line. All cells were grown at 37°C in an atmosphere with high humidity and 5% CO2. B-cells were subcultured at a concentration of 10^6^ cells/ml a night before treatments. 1,5 x 10^6^/ml cells were treated for 24 hrs by reagents as indicated. NPC cells were seeded on a 12 well plate 24hr before treatment to achieve 90% confluency (ca 0,76x106/well) at the day of treatment. Cells were treated by 10 mM SCFA solutions for 24hr. Cell viability was measured by counting live cells with the red coloured exclusion dye, erythrosin B [30].

### RNA isolation, cDNA synthesis, and PCR

RNA was purified with RNeasy Mini Kit (Qiagen, USA). Reverse transcription was performed using RevertAid First Strand cDNA Synthesis Kit (Thermo scientific, EU). Taq DNA Polymerase, Recombinant (Thermo scientific, Sweden) wss employed for amplification. The results were analyzed by agarose gel electrophoresis and recorded by system Gel Doc^™^ XR ChemiDoc^™^ XRS—Bio-Rad using Image Lab^™^ Software 170–82657. Signal intensities was quantitated with ImageJ software and normalized to the housekeeping gene density as indicated.

### EBV infection of Raji B cells

EBV-containing conditioned media was collected from a gastric carcinoma AGS cell line, cultured as a monolayer, carrying recombinant EBV-GFP. Viral production in AGS cells was induced by 40h incubation with 10 mM Butyric acid. Media was centrifuged before application to Raji B cells. 1x10 ^6^ Raji cells, cultured in suspension, were pelleted by centrifugation at 1000 prm for 5 min at RT, resuspended in 2 ml virus–containing supernatant with 8 mkg/ml polybrene and incubated in 6-well plates in a humidified incubator at 37°C with 5% CO_2_ for 90 h with shaking every 24hr. Cells were harvested by centrifugation at 1000 rpm for 5 min at RT and analysed using FACSCalibur flow cytometer (BD Biosciences) using the CellQuest Pro program.

### Western blot analysis

Cells were lysed in the presence of 1% NP-40 detergent and protein samples were prepared as described previously [31]. Protein contents were equalized after measurement with Bradford [32]. Total cell extracts were separated on 4–10% polyacrylamide gels (Bio-Rad) with sodium dodecyl sulfate (SDS) and were transferred to nitrocellulose membranes (Bio-Rad). Protein transfer was visualized by PonceauS staining [33]. Western blot signal intensities were quantitated with ImageJ software and normalized to the actin band density. Images of membranes were recorded by the system Gel Doc^™^ XR ChemiDoc^™^ XRS—Bio-Rad using Image Lab^™^ Software 170–82657. The following antibodies were used at 1:1000 dilutions if not otherwise noted: anti-PARP (AB3565, Millipore), anti-Phospho-IkappaBalpha Ser 32/36 (5A5, Cell Signaling), anti-IkappaB-alpha (C-21), anti-MCT4 (H-90), anti-beta-actin (I-19), Santa Cruz Biotechnology.

### Human IL-8/CXCL8 protein secretion

This was analyzed with a DuoSet ELISA kit, catalog# DY208-05, R&D Systems, accordingly to a manufacturer protocol.

### Annexin V and Propidium Iodine staining

This was analyzed with Annexin V-FITC Apoptosis Detection Kit, catalog#:K101-25, Biovision, accordingly to the manufacturer´s protocol.

Both, for ELISA and Annexin V staining, Raji or C666-1 cells were plated on a 6 well plate at 1x10^6^ /well overnight before treatment. Cells were left untreated or exposed to either 10mM HCl or 10mM butyric acid, for 18hr, if not pointed otherwise.

### Scratch wound assay

EBV positive and negative epithelial cell lines, CNE2 and HONE1, were seeded in a 6 well plate for 24hr to each 90% confluency at the day of treatment. Cells were then treated with SCFAs for 24hr. A scratch with a P10 pipette tip was made at the time of treatment [34,35]. Images of scratches were recorded by Olympus CKX 41 microscope at 4x magnification and processed by a DpxView Pro [1.14.3] software at 16hr. The remaining width of the scratch gap at 16hr was normalized to the gap measured at 0 hours.

### Knock-down of butyrate transporters

siRNAs from Santa-Cruz was employed, against MCT1(sc-37235) and MCT4(sc-45892), with Lipofectamine RNAiMAX reagent, catalog#1377–100, Invitrogen, accordingly to the manufacturer´s protocol. In brief, 10nM siMCT1 or siMCT4, together with 1 ug plasmid, coding for a red fluorescent protein pTomato (cat#632532, Clontech, US), to monitor transfection efficiency, was introduced into C666-1 cells seeded one day earlier in a 6 well plate and treated 24 hrs later with either 10mM HCl or n-butyric acid.

### Library construction, sequencing and analysis

The libraries were produced from the expressed mRNAs in parental HONE1 and stably LMP2A transfected NPC cell line before and after exposure to 10mM butyric acid for 24hr. cDNA libraries were prepared by Smartseq2 [36] protocol and sequenced on Illumina HiSeq 2000 platform. QC analysis was performed on the resulting sample reads using FastQC tool [37]. The reads were mapped to the UCSC reference human genome (version hg19) using STAR mapper [38]. Gene read counts and expression level were expressed in RPKMs (reads per million mapped read per kb of transcript length) and calculated based on uniquely mapped reads using an in-house pipeline [39].

### Network enrichment analysis

The procedure of network enrichment analysis, NEA, [40] is similar to that of Gene Set Enrichment Analysis, GSEA [41]. In comparison to GSEA, NEA has the advantage of analyzing any DE genes in the global network, regardless of having a pathway/GO annotation. Another difference is that genes from the latter are analyzed in terms of network connectivity to the DE genes and do not have to be differentially expressed themselves. The software and the global interaction network for NEA were those presented by Merid et al., 2014 [42].

## Results

### Short chain fatty acids induced EBV lytic cycle in B-cell lymphoma and epithelial cancer derived cells

**Fig 1:**
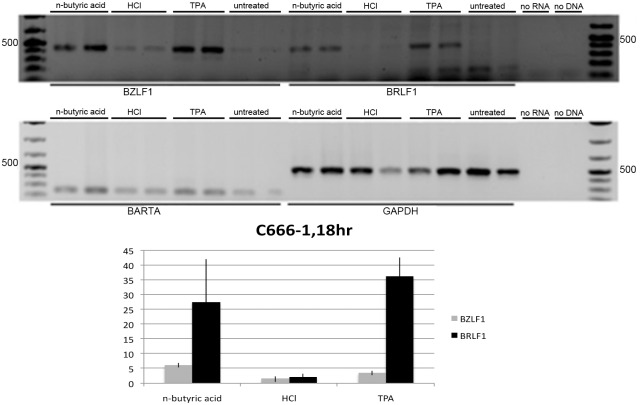
Expression of EBV lytic IE genes in Raji cells. RT-PCR analysis upon overnight treatments with 10mM n-butyric acid, 10mM HCl or 20ng/ml TPA.

**Fig 2:**
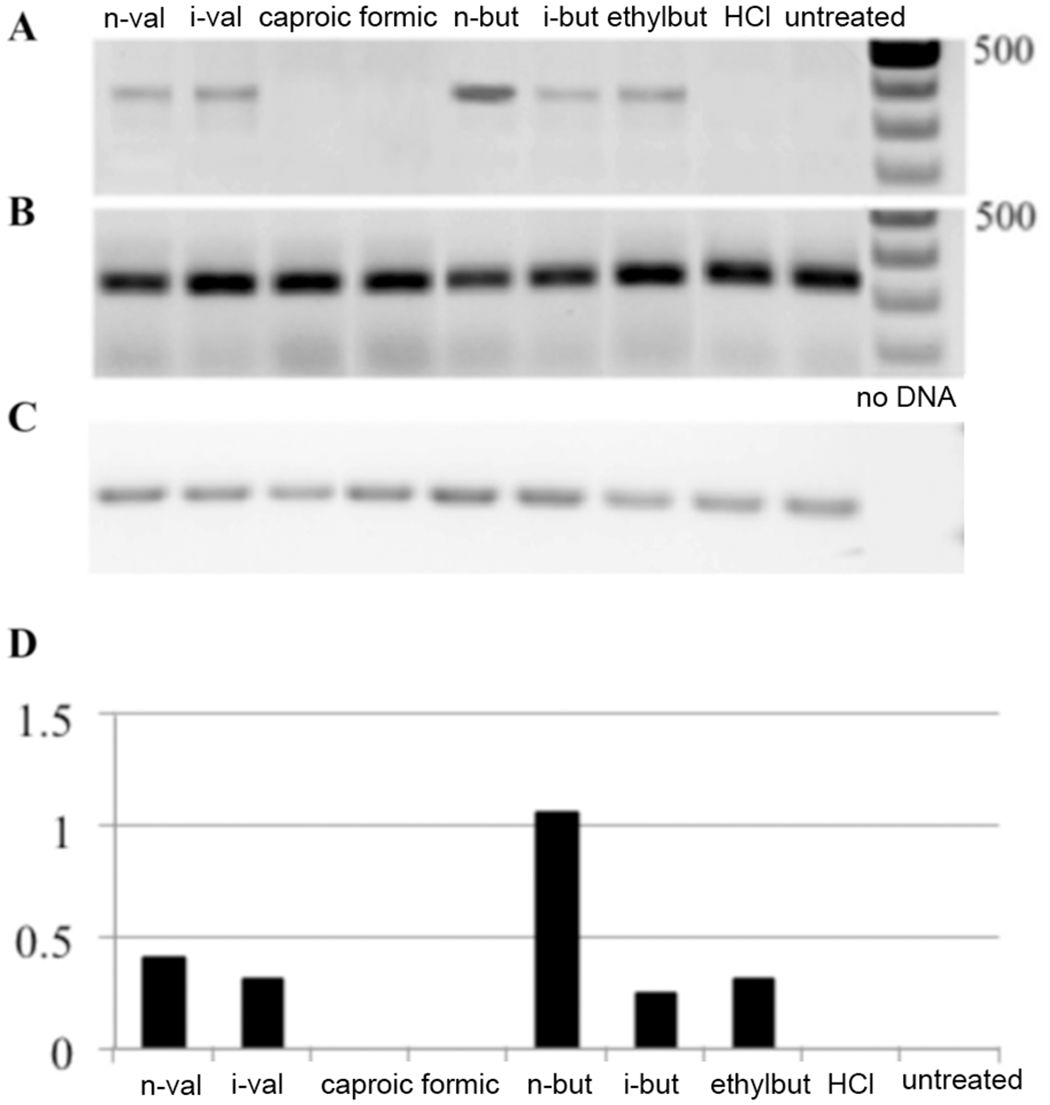
Analysis of short chain fatty acids induced BZLF1 gene expression in C666-1 NPC cells. RT-PCR analysis upon overnight treatment with 10mM of short chain fatty acids as indicated. A. BZLF1 expression. B. BART A expression. C. GAPDH expression. Cells were treated with 10mM of SCFAs as indicated or were left untreated. D. Digital analysis of data from panels A-C, demonstrating BZLF1 expression in relation to GAPDH.

**Fig 3:**
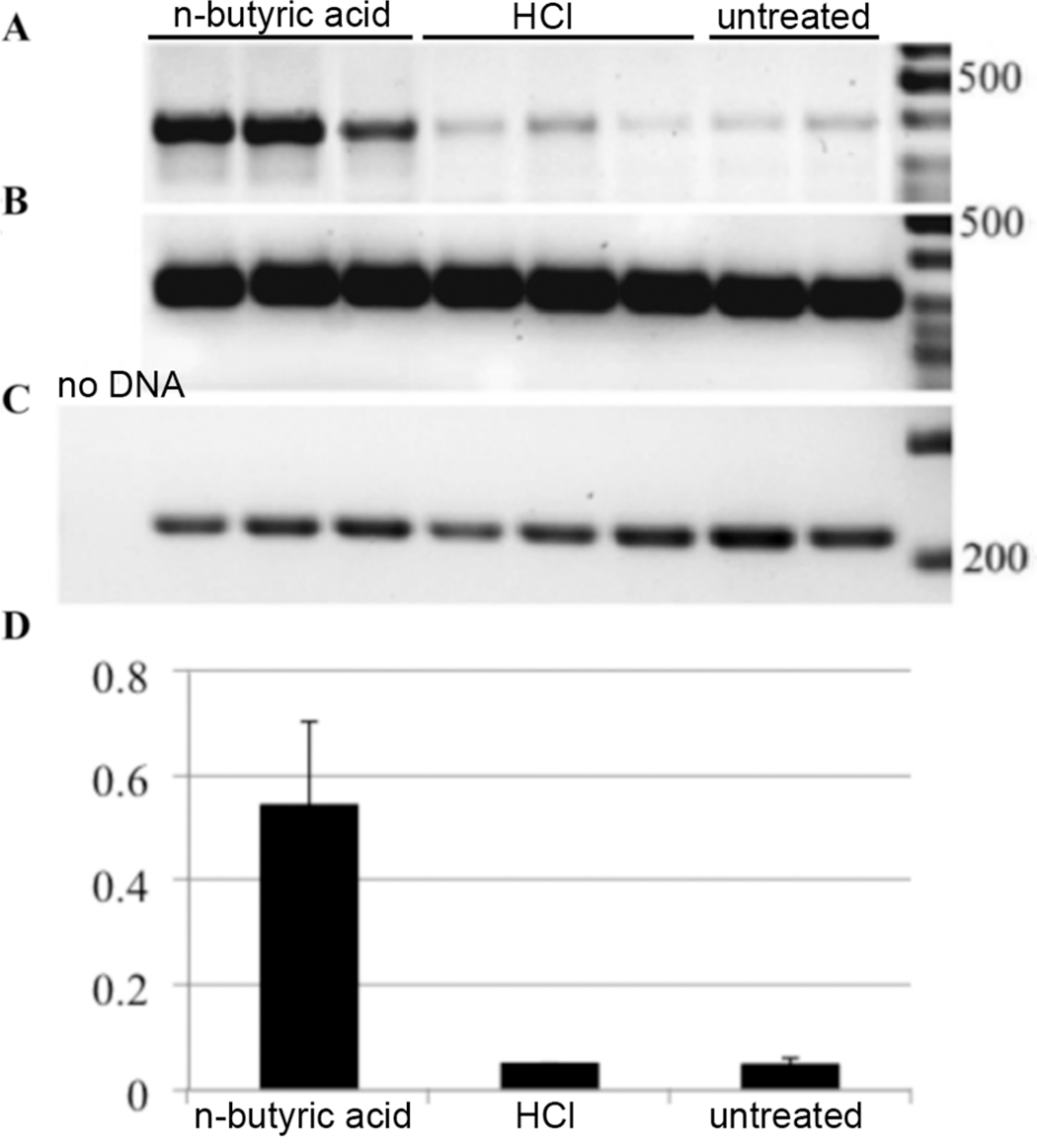
Analysis of n-butyric acid induced BZLF1 gene expression in C666-1 NPC cells. RT-PCR analysis upon treatment with 10mM of n-butyric acid or HCl. A. BZLF1 expression. B. GAPDH expression. C. BART A expression. D. Digital analysis of relative BZLF1 expression calibrated to GAPDH, calculated from data in panels A-C.

**Fig 4:**
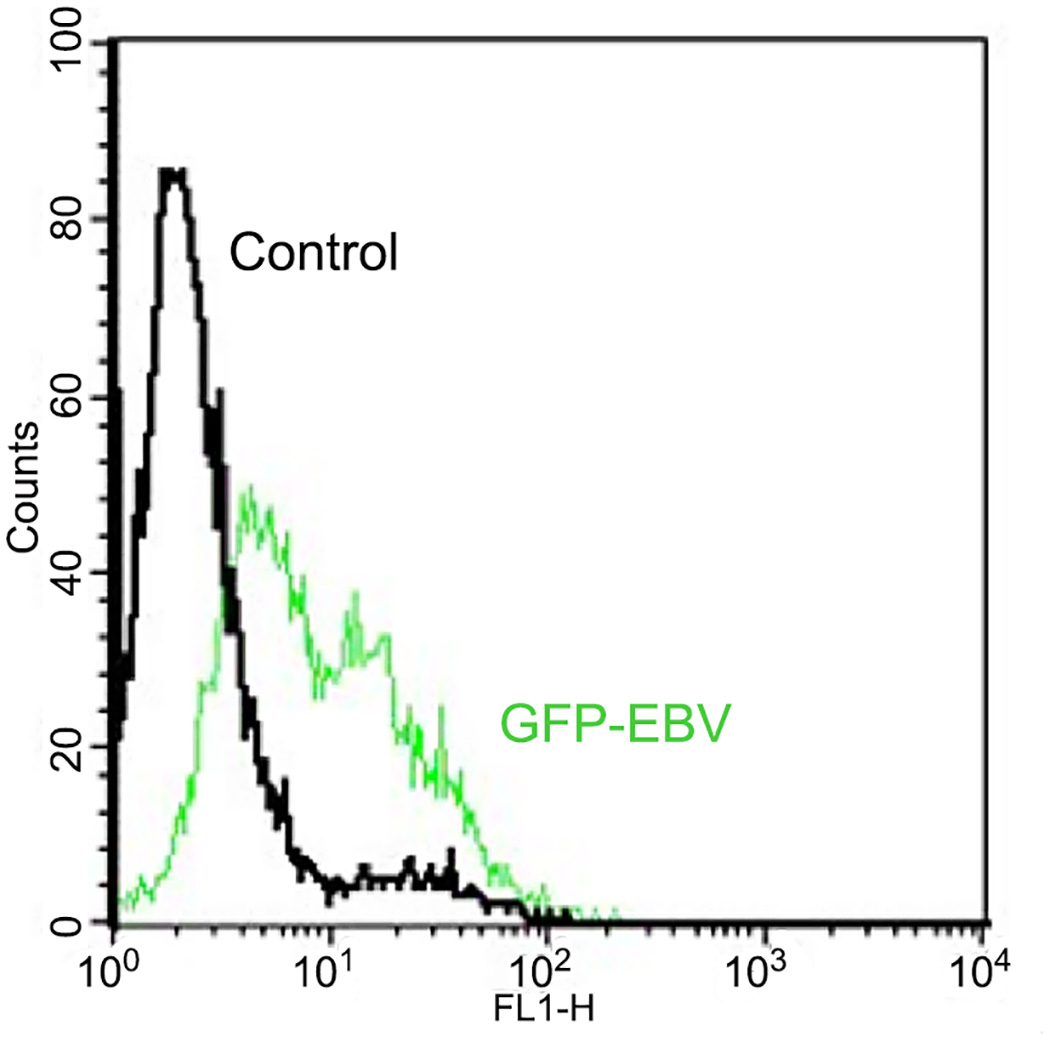
N-butyric acid induces EBV virus production. Flow cytometric analysis of GFP fluorescence in Raji cells upon superinfection with EBV-GFP recombinant virus produced by EBV-GFP carrying AGS epithelial cells, upon treatment with n-butyric acid as stated in the M&M.

We tested expression of EBV immediate early genes in Raji cells treated with n-butyric acid. In comparison to untreated Raji cells (Fig 1, lanes 7–8) all treated cells showed significant increase in expression of BZLF1 and BRLF1, the EBV immediate early genes (two upper panels, Fig 1) in relation to expression of the house-keeping GAPDH gene and an internal control for EBV latent gene expression, the EBV transcript BART A (Fig 1, the lowest panel). TPA, an established inducer of EBV lytic replication, was used as a control of induction. The observed pattern of EBV lytic genes was not due to decrease of pH due to the exposure to acids, as in cells treated with low molarity of hydrochloric acid (HCl), with comparable pK, we did not observe induction of EBV IE genes expression (Fig 1, lanes 3–4). Thus, we confirmed that 10 mM n-butyric acid induced the early phase of the lytic cycle. We then used BZLF1 expression as a read-out for testing a whole panel of SCFAs on EBV-carrying C666-1 cells. In comparison to non-treated C666-1 cells (Fig 2, lane 9) the most significant upregulation of BZLF1, was obtained with 10 mM n-butyric acid (Fig 2, lane 1). The BZLF1 response to SCFAs in C666-1 cells was highly reproducible (Fig 3). We employed one more EBV model cell line to confirm the effect of n-butyric acid on EBV lytic cycle replication. We took the advantage of a recombinant EBV virus, which also carries the gene for green fluorescent protein (GFP). We exposed this AGS cell line carrying EBV-GFP to 10mM n-butyric acid, collected supernatants and applied these to the Raji B cell line, accordingly to the protocol, described in Material and Methods. After exposure to n-butyric acid the AGS cell supernatants resulted in infection of Raji, as indicated by appearance of GFP positive Raji cells, while no infection was seen with supernatants of mock or HCl treated AGS cells (Fig 4).

### N-Butyric acid induced inflammatory response in B-cell lymphoma and epithelial cancer cells

**Fig 5:**
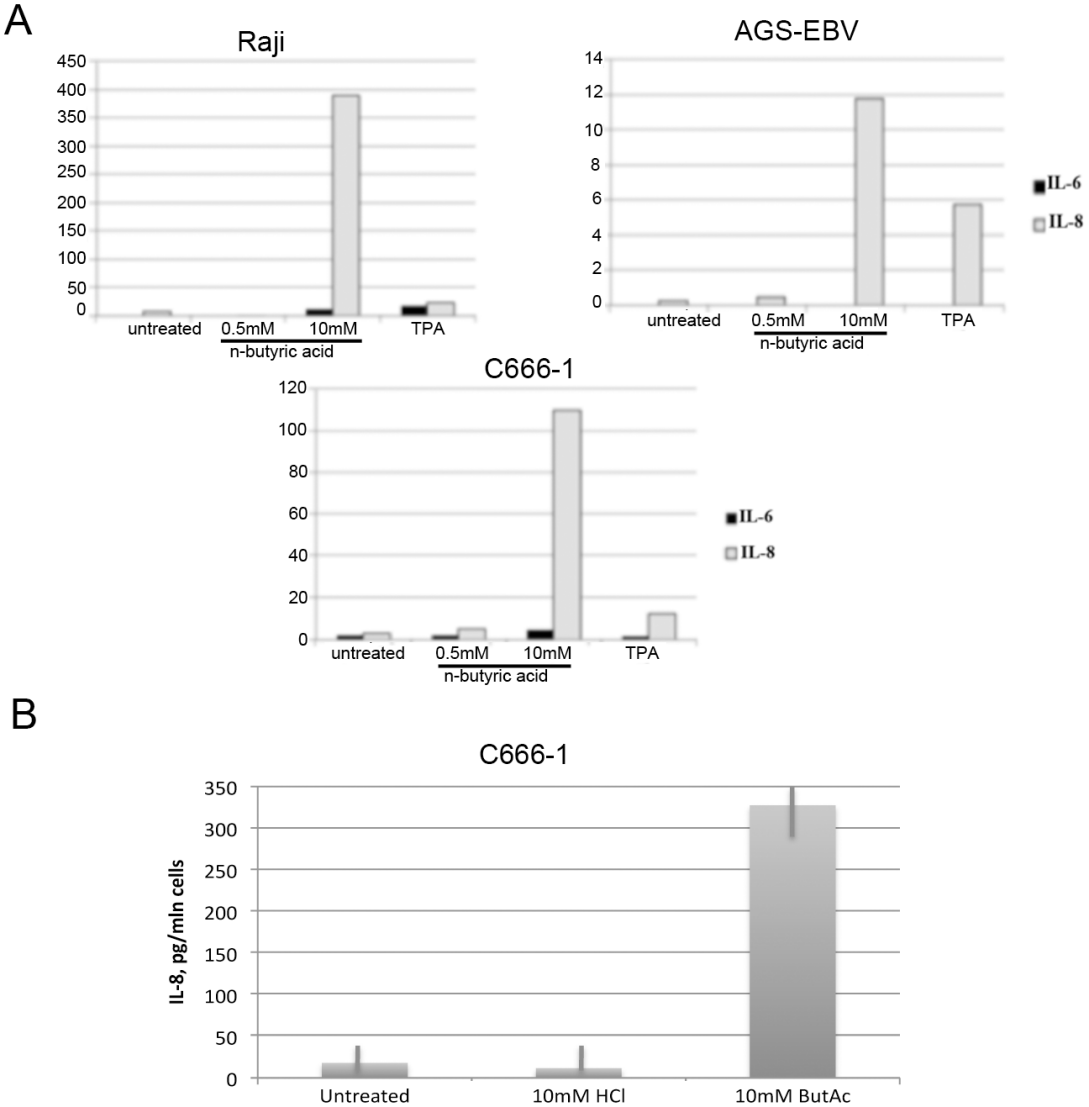
N-butyric acid induces proinflammatory cytokine expression. A. qRT-PCR analysis of Il-6 and IL-8 expression after overnight exposure to 10mM n-butyric acid. Values are adjusted to the EF1α gene expression. B. ELISA analysis of IL-8 secretion from C666-1 cells after 48hr of exposure to 10mM n-butyric acid.

**Fig 6:**
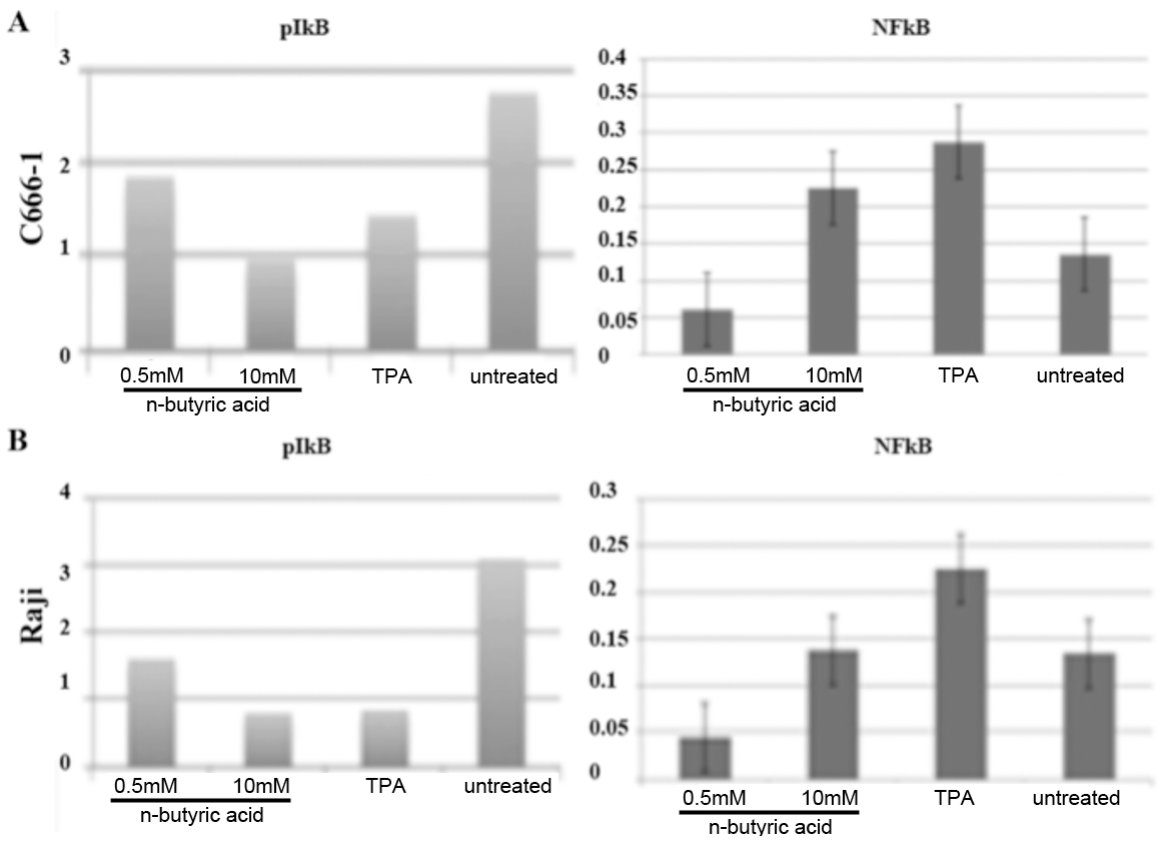
N-butyric acid induces NFkappaB signaling. Western blot analysis of phospho-IkappaB and NFkappaB expression in treated C666-1 (A) and Raji cells (B), adjusted to IkappaB and GAPDH expression, respectively.

N-Butyric acid significantly induced expression of proinflammatory cytokines IL-6 and IL-8, which we demonstrated by qRT-PCR (Fig 5A) and ELISA analysis. We performed ELISA for the strongest induced cytokine, IL-8 (Fig 5B). While upregulation of IL-6 and IL-8 genes is detectable already after overnight exposure to butyric acid in both epithelial and BL cell lines, secretion of IL-8 cytokine, is increased only in C666-1 epithelial cells after 18hr with a peak at 48hrs. To substantiate our observation of expression of the inflammatory cytokines, we analysed activation of NFkB, a crucial transcription factor responsible for the cytokine expression. We detected a decrease in the expression of the NFkB inhibitory factor, IkB, in our cell lines of epithelial and B cell origin upon treatment with n-butyric acid (Fig 6) and an increase of NFkB expression to the same level as after TPA-exposure, an established inducer of NFkB activation [19].

### N-Butyric acid induced apoptosis in epithelial and B cell lines

**Fig 7:**
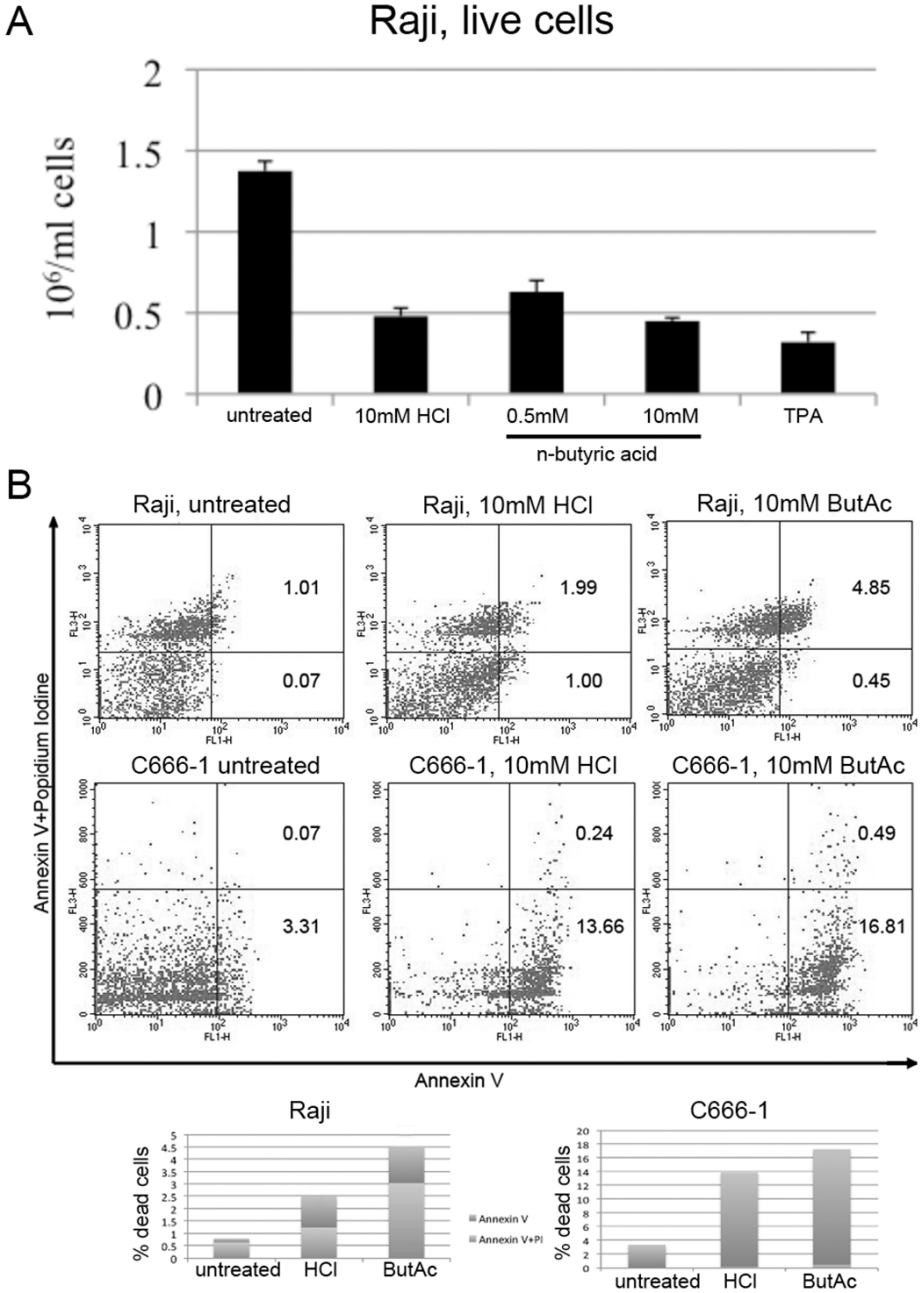
N-butyric acid induces apoptosis in Raji cells. A.The number of live cells after 24hr of treatment as indicated. B. Flow cytometry dot plot from a representative experiment. A staple diagram in the lower panel represents the apoptotic cells population (Annexin-V-positive cells+Annexin-V/propidium iodide-positive cells) for each condition.

Cell death after SCFA treatment was evaluated counting live Raji cell numbers 24hr after the treatments. Only n-butyric acid and TPA induced EBV lytic cycle, while all SCFAs treatment regimens resulted in considerable decrease in the cell numbers (Fig 7A). Apoptosis was measured by flow cytometry analysis after propidium iodide and Annexin-V-FITC labeling (Fig 7B).

**Fig 8:**
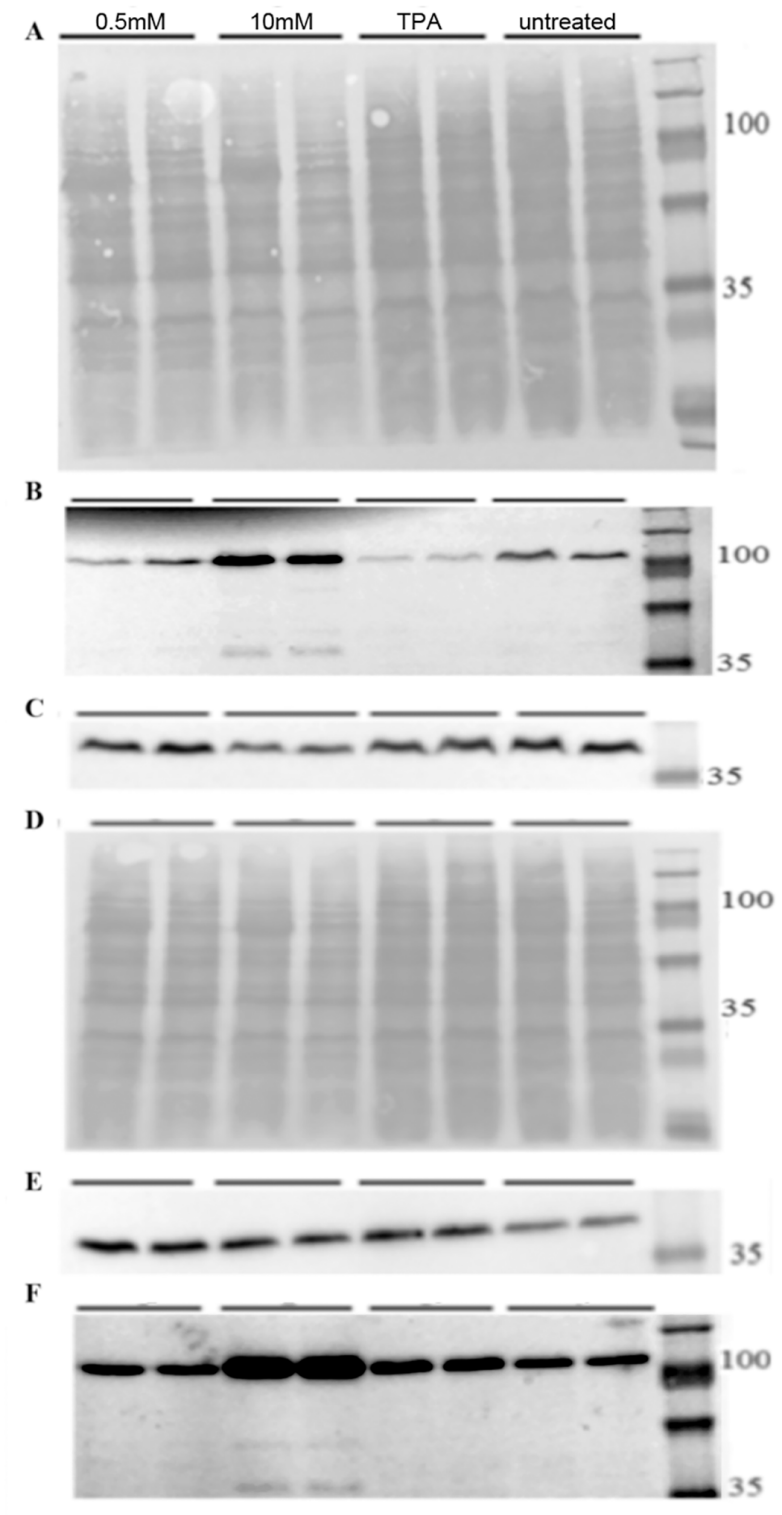
N-butyric acid induces PARP cleavage. Western blot analysis of cells treated with butyrate or TPA for 24hr, A-C) Raji cells, D-F) C666-1 cells. A,D) PonceauS staining. C,E) Actin. B,F) anti PARP Ab.

In addition, we evaluated PARP cleavage by western blot (Fig 8). Despite cell death after all treatment regimens, the PARP cleavage product—as a specific indicator of apoptosis—was detected only after 10mM n-butyric acid treatment of Raji and C666-1 cells.

### N-Butyric acid inhibited NPC cancer cells migration

**Fig 9:**
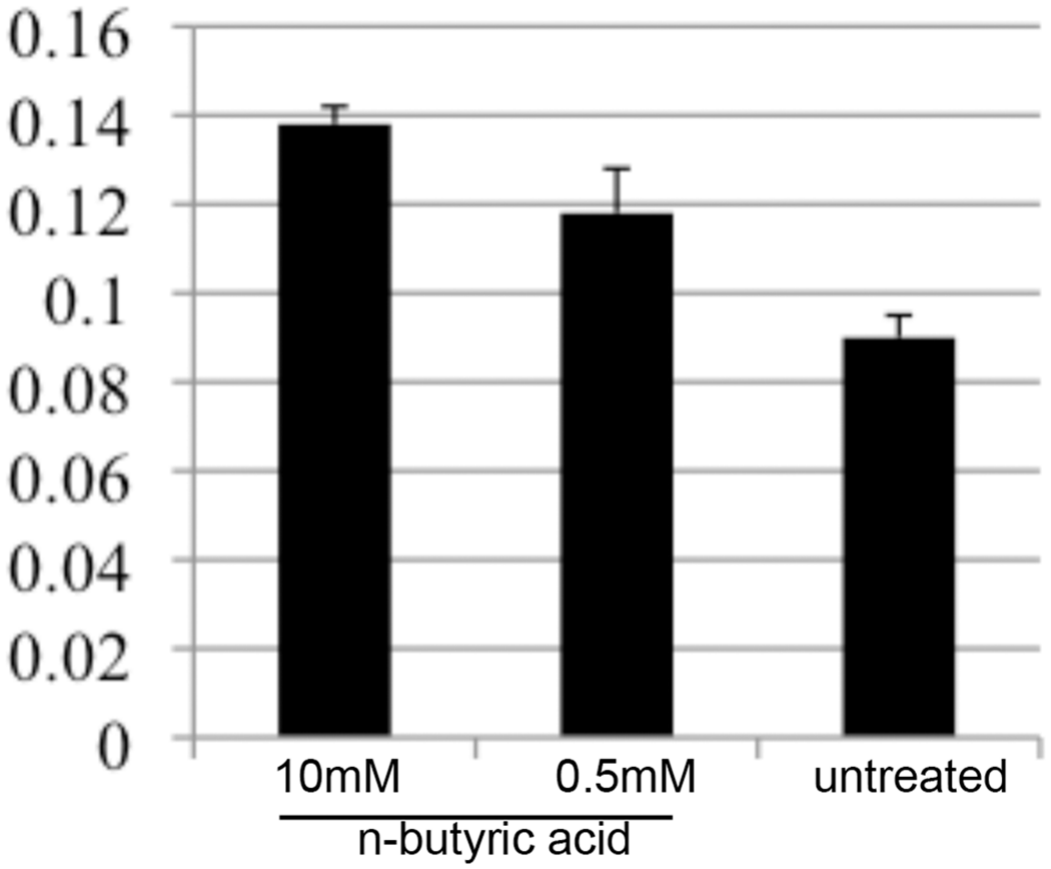
N-butyric acid inhibits migration of C666-1 NPC cells. Evaluation of the gap distance in migration assay of C666-1 NPC cells upon treatment as indicated.

To evaluate the impact of n-butyric acid on the behavior of highly migratory NPC cancer cells we analyzed C666-1 and HONE1 (data not shown) migration in a scratch wound assay. Reproducibly n-butyric acid inhibited C666-1 NPC cell migration, even at 0.5mM concentration, as judged by degree of gap closing in the scratch wound assay for C666-1 cells (Fig 9).

### Expression of n-butyric acid transporters in epithelial and B cells

**Fig 10:**
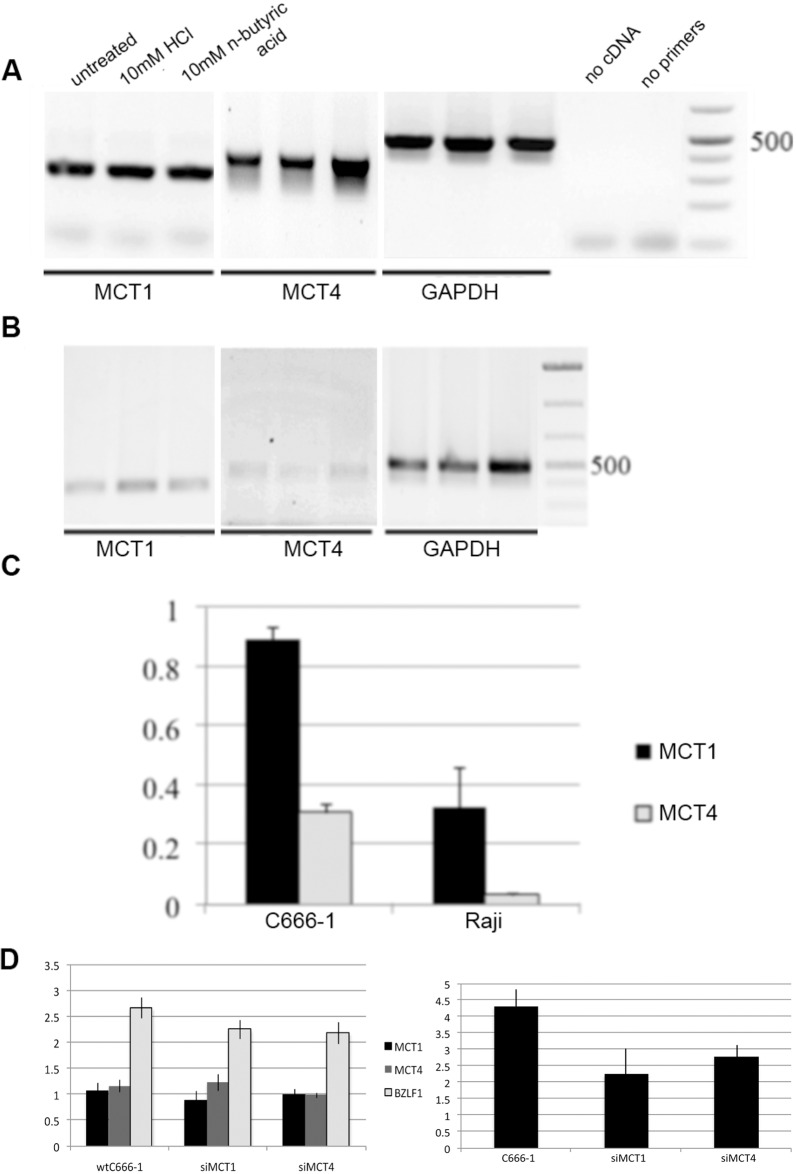
N-butyric acid enters cells through MCT1 and 4 transporters. A. RT-PCR analysis of MCT1 and 4 expression in C666-1 NPC cells upon n-butyric acid treatment during 24hr. B. RT-PCR analysis of MCT expression in Raji B cells upon different treatments during 24hr. Lanes represent the same as in A. C. Graph represents MCT1 and 4 gene expression in non treated cells, measured as pixel density and adjusted to GAPDH expression. D. The left panel. Expression of BZLF1, the viral lytic message, and cellular MCT transporters in C666-1 cells, adjusted to BART A and GAPDH, respectively, is decreased after knock-down of MCT, followed by n-butyric acid treatment. The right panel. qRT-PCR analysis of IL-8 expression in these cells. Pixel density values are compared to those after HCl treatment.

We investigated expression of MCT1 and MCT4, cellular SCFA transporters, in epithelial and B cells. We observed lower MCT1 and 4 expression in Raji B cells than in C666-1 epithelial cells (Fig 10A–10C). Treatment with n-butyric acid resulted in increase of the MCT expression in C666-1 cells, which was most evident for MCT4 (Fig 10A). We could not detect changes in MCT expression in Raji cells after treatment with n-butyric acid (Fig 10B). Activation of EBV lytic replication (BZLF1expression) decreased in response to butyric acid in cells, when MCT1 and MCT4-expression were knocked-down by siRNA (Fig 10D, the left panel). The expression of IL-8 cytokine was also decreased in these cells (Fig 10D, the right panel). This shows an important role for these transporters in butyric acid cell entrance.

### Gene expression analysis

In order to quantify changes in gene expression of NPC cells upon n-butyric acid exposure, we compared mRNA sequencing of parental HONE1 and HONE1 LMP2A transfected cells, since the LMP2A-mRNA is commonly detected in EBV associated malignancies [24]. A total of 15716 genes matching mRNA in HONE1 and 15331 genes matching those in HONE1 LMP2A cells passed preprocessing and mapping steps according to [39] and were used for the analysis of differential expression (S1 Table). We counted genes as differentially expressed when they 1) did not have exact '0' values in either column, 2) had a sum of RPKM values in two columns above 25 and 3) the RPKM values changed at least 2-fold in either direction. By using these criteria we obtained two sets of genes with altered expression (altered gene sets, AGS): 309 genes in HONE1 cells and 957 genes in LMP2A expressing HONE1 cells. These lists were analyzed for functional relations to signaling-related terms from Gene Ontology database using the method of Network Enrichment Analysis, NEA[40, 42). At the confidence threshold of false discovery rate (FDR) <0.01, we could identify in HONE1 cells four signaling pathways enriched in network connections to DE genes in HONE1 NPC cells after n-butyric acid treatment—all with highly relevant biological impact.

Thus, 424 gene members of the GO term “apoptosis” had 2834 links in total, i.e. in the global network. These genes had 627 links to the set of differentially expressed genes in our experiment (AGS), which was highly significant (Table 1).

**Table 1:** Genes expression of which changed in HONE1 cells treated with 10mM Butyric Acid for 24hr, were enriched for four different signaling-related GO terms.

Functional gene set	No. of genes in FGS	No.of links in FGS	No. of links between AGS and FGS	Network enrichment score	False discovery rate
GO_BP:CELL MIGRATION	93	662	167	11.87	5.03–03
GO_BP:APOPTOSIS	424	2834	627	11.50	5.76E-03
GO_BP:IMMUNE RESPONSE	232	1500	345	10.43	8.54E-03
GO_BP:KAPPAB_KINASE_NFKB_CASCADE	107	597	146	8.07	2.45E-02

Foot notes:

FGS–Functional Gene Set, a previously characterized group of genes with a common function.

FDR–False Discovery Rate of the network enrichment analysis

Score–the chi-squared score of network enrichment.

**Fig 11:**
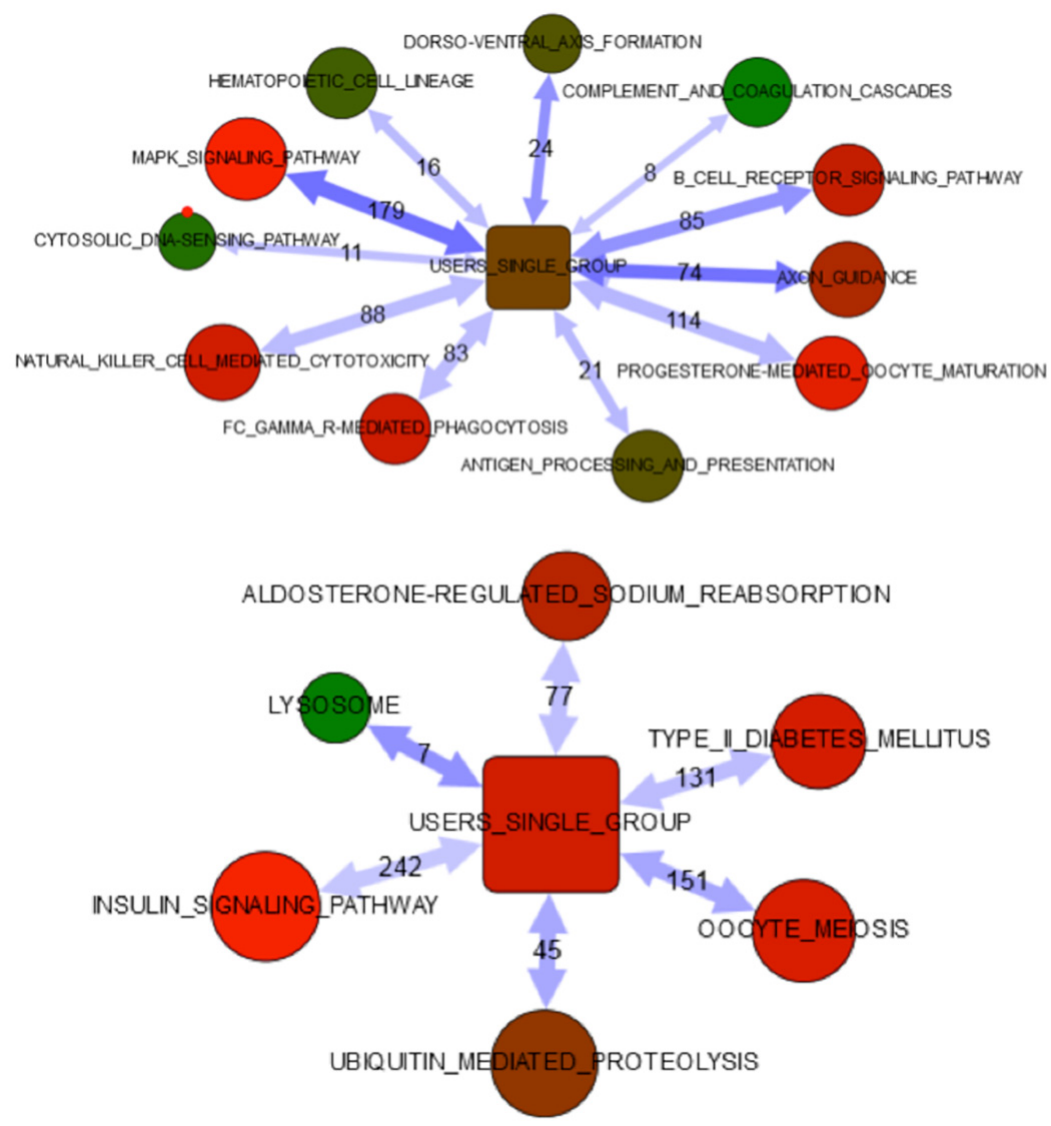
N-butyric acid affects cellular gene expression in LMP2A expressing cells. Network enrichment analysis of differentially expressed genes in HONE1 and HONE1 LMP2A cells upon n-butyric acid exposure.

By analyzing enrichment of functional relations to KEGG pathways [42], we could see up-regulation of inflammatory Toll-like receptor signaling (S2 and S3 Tables) and pathways controlling cellular motility, such as axon guidance, actin cytoskeleton, focal adhesion and adherence junction as response to butyric acid (S2 and S3 Tables). To reveal effects of LMP2A expression in HONE1 NPC cells we set the threshold for RPKM fold selection to 50. LMP2A positive cells activate fewer signaling pathways (Fig 11, compare the upper and lower bubble diagrams).

Table 2 summarizes the signaling pathways differentially enriched in the parental NPC cells and the LMP2A transfected HONE1 NPC cells in response to n-butyric acid treatment.

**Table 2:** A comparison of the differentially activated signaling pathways in LMP2A positive cells.

HONE1	HONE1+LMP2A
KEGG_04010_MAPK_SIGNALING_PATHWAY	KEGG_04142_LYSOSOME
KEGG_04360_AXON_GUIDANCE	KEGG_04114_OOCYTE_MEIOSIS
KEGG_04320_DORSO-VENTRAL_AXIS_FORMATION	KEGG_04120_UBIQUITIN_MEDIATED_PROTEOLYSIS
KEGG_04662_B_CELL_RECEPTOR_SIGNALING_PAT	EGG_04930_TYPE_II_DIABETES_MELLITUS
KEGG_04612_ANTIGEN_PROCESSING_AND_PRESEN	KEGG_04960_ALDOSTERONE-REGULATED_SODIUM_
KEGG_04640_HEMATOPOIETIC_CELL_LINEAGE	KEGG_04910_INSULIN_SIGNALING_PATHWAY
KEGG_04650_NATURAL_KILLER_CELL_MEDIATED_	
KEGG_04666_FC_GAMMA_R-MEDIATED_PHAGOCYTO	
KEGG_04623_CYTOSOLIC_DNA-SENSING_PATHWAY	
KEGG_04610_COMPLEMENT_AND_COAGULATION_CA	

## Discussion

SCFAs are important metabolic products of commensal microbiotas, e.g. in the nasopharynx or the gut. Produced at human mucosa the SCFAs have access to blood circulation and thus can also exert systemic effects. As it is known that some SCFAs have strong physiologic or metabolic effects on human host cells, it was of interest to investigate their effects on cell lines derived from cancers in human mucosal tissues. We employed latently EBV-infected cell lines both as a model and read out systems for SCFAs, as well as to evaluate the effect of SCFAs on EBV positive cells.

EBV-carrying cell lines from nasopharynx and EBV positive B cell lines were exposed to a range of physiological concentrations of a panel of SCFAs. We demonstrated that C666-1, Raji and AGS respond to SCFA by induction inflammatory genes and with induction of early steps of the EBV lytic cycle. It is known that EBV lytic replication is induced by Na-butyrate, and this effect has been linked to the effect of butyrate on histones. 10mM n-butyric acid significantly induces immediate early EBV genes BZLF1 and BRLF1 [43–45]. So did TPA, which we used as a positive control. Interestingly only n-butyric acid, but not TPA, also showed induction of apoptosis, evidenced by a decrease in the cell numbers, morphological changes and PARP cleavage. It has been suggested that EBV lytic induction is mechanistically linked to the triggering of cell death [46]. The concurrent induction of inflammation and apoptotic processes in our model systems points to different responses in two separate cell subpopulations. Although we observed upregulation of inflammatory gene expression in Raji BL cells, we could not detect IL-8 secretion from Raji cells upon exposure to butyric acid. Apparently, IL-8 mRNA produced in B cells without T cell help, would be quickly destroyed. It was more difficult to induce lytic replication in B cell lines than in epithelial cells with n-butyric acid.

In line with the our experimental data and a previous report [47], the effect of 10mM n-butyric acid on the signaling pathways regulating the migratory capacity of NPC cells was confirmed by gene expression analysis. We found that n-butyric acid inhibited migration of NPC cells independent of EBV status.

Butyric acid is actively transported in and out of cells by two specific transporters, MCT1 and MCT4 [48,49]. Epithelial cells express both and to a higher degree than the B cell line, which express mostly MCT1. The less efficient induction of EBV lytic cycle in Raji cells compared to epithelial cells upon n-butyric acid treatment correlated to differential expression of SCFA transporters in these cells. Recently, involvement of butyrate and MCT transporters in cell migration was demonstrated. Integrin b1 cooperates with MCT4 in migration of breast cancer cells [49]. Addition of butyric acid may induce MCT1 and/or MCT4 turnover and thus cause disruption of their cooperation with integrins in epithelial cells. A recent demonstration of the importance of proper spatial organization of cell surface receptors [50] corroborates our suggestion. Integrins a6b4, with which MCT1 and/or MCT4 may interact, were reported to contribute to NPC cell motility [51]. Interaction with butyric acid transporters MCT1 and MCT4 may increase the insight into a6b4 mediated migration.

The analysis of gene expression profiles of n-butyric acid treated and non treated HONE1 NPC cell lines, confirmed the significance of the cell signaling pathways studied, as major effects of the n-butyric acid response.

Virus-targeted lytic induction in EBV-associated malignancies may facilitate antiviral treatment and has been suggested [52]. Recently it was proposed to combine gemcitabine and valproic acid [53]. Valproic acid, a small organic acid is commonly used as an anti-epileptic drug and has several known adverse effects. N-butyric acid is a physiologically occurring SCFA and can be influenced by dietary effects on the composition of the microbiome. To regulate intestinal absorption and to establish an adequate pharmacokinetic approach will be challenges. Utilizing recent improvements in technology, both tasks are within reach and may be tried in a clinical setting.

SCFAs broadly affect host cell gene expression and programs. Several of the effects of butyric acid can be linked also to phenotypes and functions important for tumor cell biology. Possible adverse effects of SCFAs, always found also in normal physiologic conditions, may depend on concentrations and context. The inhibition of specific HDACs, like HDAC1,2,3, is most efficient in inducing apoptosis. The butyric acid inhibits exactly these HDACs, which also confer the activation of EBV [54]. The balance and level of microbial metabolites such as butyric acid may turn out to play a role in disease risks and progression. This can be influenced by dietary effects on the composition of the microflora.

## Supporting Information

S1 Table Differential gene expression after n-butyric acid treatment. (XLSX)

S2 Table KEGG pathways in HONE1 NPC cells. (DOCX)

S3 Table KEGG pathways in HONE1 LMP2A NPC cells. (DOCX)

## References

[1] Salyers AA, West SE, Vercellotti JR, Wilkins TD. Fermentation of mucins and plant polysaccharides by anaerobic bacteria from the human colon. Appl Environ Microbiol. 1977 11; 34(5): 529–533. 563214 10.1128/aem.34.5.529-533.1977 PMC242695

[2] Hill MJ. Bacterial fermentation of complex carbohydrate in the human colon. Eur J Cancer Prev. 1995 10; 4(5): 353–8. 7496323 10.1097/00008469-199510000-00004

[3] Cummings JH. Microbial Digestion of Complex Carbohydrates in Man. Proceedings of the Nutrition Society. 1984 1; 43(1): 35–44. 6709634 10.1079/pns19840025

[4] Cummings JH, Pomare EW, Branch WJ, Naylor CP, Macfarlane GT. Short chain fatty acids in human large intestine, portal, hepatic and venous blood. Gut. 1987; 28: 1221–1227. 3678950 10.1136/gut.28.10.1221 PMC1433442

[5] Topping DL, Clifton PM. Short-Chain Fatty Acids and Human Colonic Function: Roles of Resistant Starch and Nonstarch Polysaccharides. Physiological Reviews. 2001 7; 81(3): 1031–64. 11427691 10.1152/physrev.2001.81.3.1031

[6] Layden BT, Angueira AR, Brodsky M, Durai V, Lowe WL Jr. Short chain fatty acids and their receptors: new metabolic targets Translational Research. 2013 3; 161(3): 131–40. 10.1016/j.trsl.2012.10.007 23146568

[7] Holzapfela WH, Haberera P, Snelb J, Schillingera U, Huis in't Veldb JHJ. Overview of gut flora and probiotics. International Journal of Food Microbiology. 1998 5 26; 41(2): 85–101. 9704859 10.1016/s0168-1605(98)00044-0

[8] Sakata T, Yajima T. Influence of short chain fatty acids on the epithelial cell division of digestive tract. Quarterly Journal of Experimental Physiology. 1984 7; 69(3): 639–48. 6382381 10.1113/expphysiol.1984.sp002850

[9] Smitha JG, Yokoyamab WH, Germana JB. Butyric Acid from the Diet: Actions at the Level of Gene Expression. Critical Reviews in Food Science and Nutrition. 1998 5; 38(4): 259–97. 9626487 10.1080/10408699891274200

[10] Natarajan N, Pluznick PL. From microbe to man: the role of microbial short chain fatty acid metabolites in host cell biology. American Journal of Physiology—Cell Physiology. 2014 12; 307(11): 979–985. 10.1152/ajpcell.00228.2014 PMC5243208 25273884

[11] Kanauchi O, Fujiyama Y, Mitsuyama K, Araki Y, Ishii T, Nakamura T, et al Increased growth of Bifidobacterium and Eubacterium by germinated barley foodstuff, accompanied by enhanced butyrate production in healthy volunteers. Int J Mol Med. 1999 2; 3(2): 175–9. 9917526 10.3892/ijmm.3.2.175

[12] Tuohy KM, Rouzaud GCM, Bruck WM, Gibson GR. Modulation of the Human Gut Microbiota Towards Improved Health Using Prebiotics—assessment of Efficacy. Current Pharmaceutical Design. 2005; 11(1): 75–90. 15638753 10.2174/1381612053382331

[13] Lin HV, Frassetto A, Kowalik EJ, Nawrocki AR, Lu MM, Kosinski JR, et al Butyrate and Propionate Protect against Diet-Induced Obesity and Regulate Gut Hormones via Free Fatty Acid Receptor 3-Independent Mechanisms. PLoS One. 2012; 7(4): e35240 10.1371/journal.pone.0035240 22506074 PMC3323649

[14] Blottierea HM, Buechera B, Galmichea J-P, Cherbuta C. Molecular analysis of the effect of short-chain fatty acids on intestinal cell proliferation. Proceedings of the Nutrition Society. Proceedings of the Nutrition Society. 2003 2; 62(1): 101–6. 12740064 10.1079/PNS2002215

[15] Waldecker M, Kautenburger T, Daumann H, Busch C, Schrenk D. Inhibition of histone-deacetylase activity by short-chain fatty acids and some polyphenol metabolites formed in the colon. The Journal of Nutritional Biochemistry. 2008 9; 19(9): 587–93. 18061431 10.1016/j.jnutbio.2007.08.002

[16] Thiagalingam S, Cheng K-H, Lee HJ, Mineva N, Thiagalingam A, Ponte JF. Histone Deacetylases: Unique Players in Shaping the Epigenetic Histone Code. Ann N Y Acad Sci. 2003 3; 983: 84–100. 12724214 10.1111/j.1749-6632.2003.tb05964.x

[17] Zhang Y, Jones C. The Bovine Herpesvirus 1 Immediate-Early Protein (bICP0) Associates with Histone Deacetylase 1 To Activate Transcription. J Virol. 2001 10; 75(20): 9571–8. 11559788 10.1128/JVI.75.20.9571-9578.2001 PMC114527

[18] Hsu CH, Hergenhahn M, Chuang SE, Yeh PY, Wu TC, Gao M, et al Induction of Epstein-Barr virus (EBV) reactivation in Raji cells by doxorubicin and cisplatin. Anticancer Res. 2002 Nov-Dec; 22(6C): 4065–71. 12553034

[19] Feng WH, Hong G, Delecluse HJ, Kenney SC. Lytic Induction Therapy for Epstein-Barr Virus-Positive B-Cell Lymphomas. J. Virol. 2004; 78(4): 1893–1902. 14747554 10.1128/JVI.78.4.1893-1902.2004 PMC369434

[20] Westphal EM, Blackstock W, Feng W, Israel B, Kenney SC. Activation of Lytic Epstein-Barr Virus (EBV) Infection by Radiation and Sodium Butyrate in Vitro and in Vivo: A Potential Method for Treating EBV-positive Malignancies. Cancer Res October. 2000; 60(20): 5781–8. 11059774

[21] Blazar B, Patarroyo M, Klein E, Klein G. Increased sensitivity of human lymphoid lines to natural killer cells after induction of the Epstein-Barr viral cycle by superinfection or sodium butyrate. J Exp Med. 1980 3; 151(3): 614–627. 6244358 10.1084/jem.151.3.614 PMC2185807

[22] Quinlivan EB, Holley-Guthrie EA, Norris M, Gutsch D, Bachenheimer SL, Kenney SC. Direct BRLF1 binding is required for cooperative BZLF1/BRLF1 activation of the Epstein–Barr virus early promoter, BMRF1. Nucleic Acids Res. 1993 4; 21(8): 1999–2007. 8393562 10.1093/nar/21.8.1999 PMC309443

[23] Binne UK, Amon W, Farrell PJ. Promoter sequences required for reactivation of Epstein-Barr virus from latency. J. Virol. 1996; 70(6): 3894–3901. 12239304 10.1128/JVI.76.20.10282-10289.2002 PMC136560

[24] Cheung ST, Huang DP, Hui AB, Lo KW, Ko CW, Tsang YS, et al Nasopharyngeal carcinoma cell line (C666-1) consistently harbouring Epstein-Barr virus. Int J Cancer. 1999 9; 83(1): 121–6. 10449618 10.1002/(sici)1097-0215(19990924)83:1<121::aid-ijc21>3.0.co;2-f

[25] Barranco SC, Townsend CM Jr, Casartelli C, Macik BG, Burger NL, Boerwinkle WR, et al Establishment and characterization of an in vitro model system for human adenocarcinoma of the stomach. Cancer Res. 1983 4; 43(4): 1703–9. 6831414

[26] Yao K, Zhang HY, Zhu HC, Wang FX, Li GY, Wen DS, et al Establishment and characterization of two epithelial tumor cell lines (HNE-1 and HONE-1) latently infected with Epstein-barr virus and derived from nasopharyngeal carcinomas. International Journal of Cancer. 1990 1; 45(1): 83–9. 2153642 10.1002/ijc.2910450116

[27] Sizhong Z, Xiukung G, Yi Z. Cytogenetic studies on an epithelial cell line derived from poorly differentiated nasopharyngeal carcinoma. Int. J. Cancer. 1983; 31: 587–590. 6852976 10.1002/ijc.2910310509

[28] Epstein MA, Achong BG, Barr YM, Zajac B, Henle G, Henle W. Morphological and virological investigations on cultured Burkitt tumor lymphoblasts (strain Raji). J Natl Cancer Inst. 1966 10; 37(4): 547–59. 4288580

[29] Epstein MA, Barr YM. Characteristics and mode of growth of tissue culture strain (EB1) of human lymphoblasts from Burkitt's lymphoma. J. Natl. Cancer Inst. 1965; 34: 231–240. 14293790 10.1093/jnci/34.2.231

[30] Krause AW, Carley WW, Webb WW. Fluorescent erythrosin B is preferable to trypan blue as a vital exclusion dye for mammalian cells in monolayer culture. J Histochem Cytochem. 1984 10; 32(10): 1084–90. 6090533 10.1177/32.10.6090533

[31] Helenius A, Simons K. Solubilization of membranes by detergents. Biochim. Biophys. Acta. 1975; 415: 29–79. 1091302 10.1016/0304-4157(75)90016-7

[32] Bradford MM. A rapid and sensitive method for the quantitation of microgram quantities of protein utilizing the principle of protein-dye binding. Analytical Biochemistry. 1976 5; 72: 248–54. 942051 10.1016/0003-2697(76)90527-3

[33] Burnette WN. Western blotting': electrophoretic transfer of proteins from sodium dodecyl sulfate—polyacrylamide gels to unmodified nitrocellulose and radiographic detection with antibody and radioiodinated protein A. Analytical Biochemistry. 1981; 112 (2): 195–203. 6266278 10.1016/0003-2697(81)90281-5

[34] Friedl P, Hegerfeldt Y, Tusch M. Collective cell migration in morphogenesis and cancer. Int. J. Dev. Biol. 2004; 48: 441–449. 15349818 10.1387/ijdb.041821pf

[35] Yarrow JC, Perlman ZC, Westwood N, Mitchison TJ. A high-throughput cell migration assay using scratch wound healing, a comparison of image-based readout methods. BMC Biotechnol. 2004; 4: 21 15357872 10.1186/1472-6750-4-21 PMC521074

[36] Picelli S, Bjorklund AK, Faridani OR, Sagasser S, Winberg G, Sandberg R. Smart-seq2 for sensitive full-length transcriptome profiling in single cells. Nat Meth. 2013; 10(11): 1096–1098. 10.1038/nmeth.2639 24056875

[37] Andrews S. FastQC: a quality control tool for high throughput sequence data. 2010. Available online at: http://www.bioinformatics.babraham.ac.uk/projects/fastqc

[38] Dobin A, Davis CA, Schlesinger F, Drenkow J, Zaleski C, Jha S, et al STAR: ultrafast universal RNA-seq aligner. Bioinformatics. 2013; 29(1): 15–21. 10.1093/bioinformatics/bts635 23104886 PMC3530905

[39] Sandberg R. RPKM calculations protocol. 2015. Available online at: http://sandberg.cmb.ki.se/rnaseq/

[40] Alexeyenko A, Lee W, Pernemalm M, Guegan J, Dessen P, Lazar V, et al 2012 Network enrichment analysis: extension of gene-set enrichment analysis to gene networks. BMC Bioinformatics 13, 226 10.1186/1471-2105-13-226 22966941 PMC3505158

[41] Subramanian A, Tamayo P, Mootha VK, Mukherjee S, Ebert BL, Gillette MA, et al Gene set enrichment analysis: a knowledge-based approach for interpreting genome-wide expression profiles. Proc Natl Acad Sci U S A. 2005 10; 102(43): 15545–50. 16199517 10.1073/pnas.0506580102 PMC1239896

[42] Merid SK, Goranskaya D, Alexeyenko A. Distinguishing between driver and passenger mutations in individual cancer genomes by network enrichment analysis. BMC Bioinformatics. 2014 9; 15: 308 10.1186/1471-2105-15-308 25236784 PMC4262241

[43] Vinolo MA, Rodrigues HG, Renato T, Curi N, Curi R. Regulation of Inflammation by Short Chain Fatty Acids. Nutrients. 2011 10; 3(10): 858–876. 10.3390/nu3100858 22254083 PMC3257741

[44] Davie JR. Inhibition of histone deacetylase activity by butyrate. J Nutr. 2003 7; 133(7 Suppl): 2485S–2493S. 12840228 10.1093/jn/133.7.2485S

[45] Imai K, Inoue H, Tamura M, Cueno ME, Inoue H, Takeichi O, et al The periodontal pathogen Porphyromonas gingivalis induces the Epstein-Barr virus lytic switch transactivator ZEBRA by histone modification. Biochimie. 2012 3; 94(3): 839–46. 10.1016/j.biochi.2011.12.001 22178321

[46] Ruemmele FM, Schwartz S, Seidman EG, Dionne S, Levy E, Lentze MJ. Butyrate induced Caco-2 cell apoptosis is mediated via mitochondrial pathway. Gut 2003; 52(1): 94–100. 12477768 10.1136/gut.52.1.94 PMC1773532

[47] Wolf H, Bogedain C, Schwarzmann F. Epstein-Barr virus and its interaction with the host. Intervirology. 1993; 35(1–4): 26–39. 8407248 10.1159/000150293

[48] De Saedeleer CJ, Porporato PE, Copetti T, Perez-Escuredo J, Payen VL, Brisson L, et al Glucose deprivation increases monocarboxylate transporter 1 (MCT1) expression and MCT1-dependent tumor cell migration. Oncogene. 2014 7; 33(31): 4060–8. 10.1038/onc.2013.454 24166504

[49] Gallagher SM, Castorino JJ, Philp NJ. Interaction of monocarboxylate transporter 4 with beta1-integrin and its role in cell migration. Am J Physiol Cell Physiol. 2009 3; 296(3): 414–21. 10.1152/ajpcell.00430.2008 PMC2660264 19073896

[50] Shaw A, Lundin V, Petrova E, Fördös F, Benson E, Al-Amin A, et al Spatial control of membrane receptor function using ligand nanocalipers. Nat Methods. 2014 8; 11(8): 841–6. 10.1038/nmeth.3025 24997862

[51] Zhou X, Matskova L, Rathje LZ, Xiao X, Gish G, Werner M, et al SYK interaction with ITGb4 suppressed by Epstein-Barr virus LMP2A modulates migration and invasion of nasopharyngeal carcinoma cells. Oncogene. 2014 12: 4491–9. 10.1038/onc.2014.380 25531330

[52] Mentzer SJ, Perrine SP, Faller DV. Epstein—Barr virus post-transplant lymphoproliferative disease and virus-specific therapy: pharmacological re-activation of viral target genes with arginine butyrate. Transpl Infect Dis. 2001 9;3(3):177–85. 11493400 10.1034/j.1399-3062.2001.003003177.x

[53] Stoker SD, Novalić Z, Wildeman MA, Huitema AD, Verkuijlen SA, Juwana H, et al Epstein-Barr virus-targeted therapy in nasopharyngeal carcinoma. J Cancer Res Clin Oncol. 2015 10;141(10):1845–57. 10.1007/s00432-015-1969-3 25920375 PMC11823716

[54] Hui KF, Cheung AK, Choi CK, Yeung PL, Middeldorp JM, Lung ML, et al Inhibition of class I histone deacetylases by romidepsin potently induces Epstein-Barr virus lytic cycle and mediates enhanced cell death with ganciclovir. Int J Cancer. 2016 1 1;138(1):125–36. 10.1002/ijc.29698 26205347

