# Supplementary material for: Short Chain Fatty Acids (SCFA) Reprogram Gene Expression in Human Malignant Epithelial and Lymphoid Cells
Source: PLoS One. 2016 Jul 21;11(7):e0154102. doi: 10.1371/journal.pone.0154102 (PMC4956219; doi:10.1371/journal.pone.0154102)
Supplement: S2 Table — (DOCX) [file pone.0154102.s002.docx]

**S2 Table. KEGG pathway analysis of differentially expressed genes in HONE1 NPC cells.** Two sets of genes with altered expression (altered gene sets, AGS): 309 genes in HONE1 cells were analyzed in regard to functional relations to KEGG pathways. FGS – Functional Gene Set, a previously characterized group of genes with a common function. FDR – False Discovery Rate of the network enrichment analysis. Score –the chi-squared score of network enrichment.

HONE1 parental, No. of AGS-309, No. of links AGS-960

| **Functional gene set** | **No.** o**f genes in FGS** | **No of links in FGS** | **No of links between AGSand FGS** | **Network enrichment score** | **False discovery rate** |
| --- | --- | --- | --- | --- | --- |
| KEGG_04620_TOLL-LIKE_RECEPTOR_SIGNALING_ | 108 | 2841 | 111 | 60.20 | 0.000000E+00 |
| KEGG_04621_NOD-LIKE_RECEPTOR_SIGNALING_P | 38 | 705 | 41 | 56.82 | 0.000000E+00 |
| KEGG_04622_RIG-I-LIKE_RECEPTOR_SIGNALING | 38 | 760 | 40 | 45.18 | 4.131720E-10 |
| KEGG_04660_T_CELL_RECEPTOR_SIGNALING_PAT | 111 | 3641 | 121 | 38.79 | 8.124233E-09 |
| KEGG_04510_FOCAL_ADHESION | 201 | 4908 | 150 | 34.53 | 5.804280E-08 |
| KEGG_04110_CELL_CYCLE | 116 | 664 | 33 | 32.88 | 1.124988E-07 |
| KEGG_04010_MAPK_SIGNALING_PATHWAY | 282 | 6266 | 179 | 30.19 | 3.863310E-07 |
| KEGG_04360_AXON_GUIDANCE | 132 | 2094 | 74 | 29.38 | 4.842113E-07 |
| KEGG_04012_ERBB_SIGNALING_PATHWAY | 91 | 3673 | 115 | 29.26 | 4.842113E-07 |
| KEGG_04115_P53_SIGNALING_PATHWAY | 68 | 499 | 26 | 28.74 | 5.723343E-07 |
| KEGG_04664_FC_EPSILON_RI_SIGNALING_PATHW | 81 | 4165 | 126 | 27.73 | 8.749200E-07 |
| KEGG_04722_NEUROTROPHIN_SIGNALING_PATHWA | 121 | 4192 | 120 | 20.41 | 3.308762E-05 |
| KEGG_04370_VEGF_SIGNALING_PATHWAY | 79 | 4213 | 119 | 18.93 | 6.693986E-05 |
| KEGG_04350_TGF-BETA_SIGNALING_PATHWAY | 86 | 983 | 37 | 17.94 | 1.049260E-04 |
| KEGG_04320_DORSO-VENTRAL_AXIS_FORMATION | 23 | 572 | 24 | 15.84 | 2.978903E-04 |
| KEGG_04662_B_CELL_RECEPTOR_SIGNALING_PAT | 86 | 2923 | 85 | 15.60 | 3.181265E-04 |
| KEGG_04150_MTOR_SIGNALING_PATHWAY | 49 | 1713 | 54 | 14.11 | 6.275005E-04 |
| KEGG_04520_ADHERENS_JUNCTION | 79 | 1717 | 54 | 13.97 | 6.394920E-04 |
| KEGG_04912_GNRH_SIGNALING_PATHWAY | 103 | 5032 | 129 | 11.63 | 2.029917E-03 |
| KEGG_04914_PROGESTERONE-MEDIATED_OOCYTE_ | 106 | 4634 | 114 | 7.62 | 1.529385E-02 |
| KEGG_04612_ANTIGEN_PROCESSING_AND_PRESEN | 88 | 615 | 21 | 7.41 | 1.662210E-02 |
| KEGG_04810_REGULATION_OF_ACTIN_CYTOSKELE | 225 | 4453 | 109 | 7.00 | 1.935046E-02 |
| KEGG_04640_HEMATOPOIETIC_CELL_LINEAGE | 78 | 443 | 16 | 6.81 | 2.011392E-02 |
| KEGG_04650_NATURAL_KILLER_CELL_MEDIATED_ | 144 | 3514 | 88 | 6.73 | 2.011392E-02 |
| KEGG_04666_FC_GAMMA_R-MEDIATED_PHAGOCYTO | 84 | 3314 | 83 | 6.35 | 2.252275E-02 |
| KEGG_04623_CYTOSOLIC_DNA-SENSING_PATHWAY | 20 | 280 | 11 | 6.05 | 2.593281E-02 |
| KEGG_04610_COMPLEMENT_AND_COAGULATION_CA | 69 | 187 | 8 | 5.55 | 3.182452E-02 |
| KEGG_04210_APOPTOSIS | 89 | 2315 | 59 | 5.10 | 3.926100E-02 |
