# Supplementary material for: Short Chain Fatty Acids (SCFA) Reprogram Gene Expression in Human Malignant Epithelial and Lymphoid Cells
Source: PLoS One. 2016 Jul 21;11(7):e0154102. doi: 10.1371/journal.pone.0154102 (PMC4956219; doi:10.1371/journal.pone.0154102)
Supplement: S3 Table — (DOCX) [file pone.0154102.s003.docx]

**S3 Table. KEGG pathway analysis of differentially expressed genes in HONE1 LMP2A NPC cells.** Two sets of genes with altered expression (altered gene sets, AGS): 957 genes in LMP2A expressing HONE1 cells were analyzed in regard to functional relations to KEGG pathways. FGS – Functional Gene Set, a previously characterized group of genes with a common function. FDR – False Discovery Rate of the network enrichment analysis. Score –the chi-squared score of network enrichment.

S3 Table. HONE1 LMP2A, No. of AGS – 957, No of links AGS – 2232

| **Functional gene set** | **No.** o**f genes in FGS** | **No.** o**f links in FGS** | **No of links between AGS and FGS** | **Network enrichment score** | **False discovery rate** |
| --- | --- | --- | --- | --- | --- |
| KEGG_04115_P53_SIGNALING_PATHWAY | 68 | 499 | 80 | 152.27 | 0.000000E+00 |
| KEGG_04110_CELL_CYCLE | 116 | 664 | 84 | 101.81 | 0.000000E+00 |
| KEGG_04350_TGF-BETA_SIGNALING_PATHWAY | 86 | 983 | 80 | 30.77 | 5.021302E-07 |
| KEGG_04510_FOCAL_ADHESION | 201 | 4908 | 289 | 24.09 | 1.269379E-05 |
| KEGG_04111_CELL_CYCLE_-_YEAST | 50 | 96 | 14 | 22.42 | 2.514130E-05 |
| KEGG_04621_NOD-LIKE_RECEPTOR_SIGNALING_P | 38 | 705 | 55 | 18.24 | 1.922636E-04 |
| KEGG_04142_LYSOSOME | 8 | 37 | 7 | 17.59 | 2.364716E-04 |
| KEGG_04660_T_CELL_RECEPTOR_SIGNALING_PAT | 111 | 3641 | 213 | 16.91 | 3.003340E-04 |
| KEGG_04622_RIG-I-LIKE_RECEPTOR_SIGNALING | 38 | 760 | 57 | 16.31 | 3.708819E-04 |
| KEGG_04520_ADHERENS_JUNCTION | 79 | 1717 | 110 | 15.35 | 5.481532E-04 |
| KEGG_04722_NEUROTROPHIN_SIGNALING_PATHWA | 121 | 4192 | 232 | 11.83 | 2.517551E-03 |
| KEGG_04114_OOCYTE_MEIOSIS | 90 | 2610 | 151 | 11.03 | 3.434705E-03 |
| KEGG_04120_UBIQUITIN_MEDIATED_PROTEOLYSI | 121 | 629 | 45 | 10.63 | 4.029963E-03 |
| KEGG_04914_PROGESTERONE-MEDIATED_OOCYTE_ | 106 | 4634 | 251 | 10.46 | 4.210725E-03 |
| KEGG_04370_VEGF_SIGNALING_PATHWAY | 79 | 4213 | 230 | 10.34 | 4.284243E-03 |
| KEGG_04012_ERBB_SIGNALING_PATHWAY | 91 | 3673 | 201 | 9.23 | 7.134600E-03 |
| KEGG_04664_FC_EPSILON_RI_SIGNALING_PATHW | 81 | 4165 | 221 | 7.42 | 1.849315E-02 |
| KEGG_04930_TYPE_II_DIABETES_MELLITUS | 48 | 2353 | 131 | 7.02 | 2.209566E-02 |
| KEGG_04210_APOPTOSIS | 89 | 2315 | 129 | 6.96 | 2.209566E-02 |
| KEGG_04960_ALDOSTERONE-REGULATED_SODIUM_ | 34 | 1300 | 77 | 6.64 | 2.451718E-02 |
| KEGG_04912_GNRH_SIGNALING_PATHWAY | 103 | 5032 | 260 | 6.38 | 2.747628E-02 |
| KEGG_04810_REGULATION_OF_ACTIN_CYTOSKELE | 225 | 4453 | 231 | 5.96 | 3.375480E-02 |
| KEGG_04910_INSULIN_SIGNALING_PATHWAY | 144 | 4708 | 242 | 5.54 | 4.125310E-02 |
| KEGG_04150_MTOR_SIGNALING_PATHWAY | 49 | 1713 | 96 | 5.43 | 4.255791E-02 |
